# Seroprevalence and Spatial Epidemiology of Lymphatic Filariasis in American Samoa after Successful Mass Drug Administration

**DOI:** 10.1371/journal.pntd.0003297

**Published:** 2014-11-13

**Authors:** Colleen L. Lau, Kimberly Y. Won, Luke Becker, Ricardo J. Soares Magalhaes, Saipale Fuimaono, Wayne Melrose, Patrick J. Lammie, Patricia M. Graves

**Affiliations:** 1 WHO Collaborating Centre for Children's Health and Environment, Queensland Children's Medical Research Institute, The University of Queensland, Brisbane, Australia; 2 Research School of Population Health, Australian National University, Canberra, Australia; 3 Centers for Disease Control and Prevention, Division of Parasitic Diseases and Malaria, Atlanta, Georgia, United States of America; 4 WHO Collaborating Centre for Lymphatic Filariasis, Soil-Transmitted Helminths, and other Neglected Tropical Diseases, Division of Tropical Health and Medicine, James Cook University, Cairns, Australia; 5 School of Veterinary Science, The University of Queensland, Gatton, Australia; 6 American Samoa Department of Health, Pago Pago, American Samoa; University of Ghana, Ghana

## Abstract

**Background:**

As part of the Global Programme to Eliminate Lymphatic Filariasis (LF), American Samoa conducted mass drug administration (MDA) from 2000–2006, and passed transmission assessment surveys in 2011–2012. We examined the seroprevalence and spatial epidemiology of LF post-MDA to inform strategies for ongoing surveillance and to reduce resurgence risk.

**Methods:**

ELISA for LF antigen (Og4C3) and antibodies (Wb123, Bm14) were performed on a geo-referenced serum bank of 807 adults collected in 2010. Risk factors assessed for association with sero-positivity included age, sex, years lived in American Samoa, and occupation. Geographic clustering of serological indicators was investigated to identify spatial dependence and household-level clustering.

**Results:**

Og4C3 antigen of >128 units (positive) were found in 0.75% (95% CI 0.3–1.6%) of participants, and >32 units (equivocal plus positive) in 3.2% (95% CI 0.6–4.7%). Seroprevalence of Wb123 and Bm14 antibodies were 8.1% (95% CI 6.3–10.2%) and 17.9% (95% CI 15.3–20.7%) respectively. Antigen-positive individuals were identified in all ages, and antibody prevalence higher in older ages. Prevalence was higher in males, and inversely associated with years lived in American Samoa. Spatial distribution of individuals varied significantly with positive and equivocal levels of Og4C3 antigen, but not with antibodies. Using Og4C3 cutoff points of >128 units and >32 units, average cluster sizes were 1,242 m and 1,498 m, and geographical proximity of households explained 85% and 62% of the spatial variation respectively.

**Conclusions:**

High-risk populations for LF in American Samoa include adult males and recent migrants. We identified locations and estimated the size of possible residual foci of antigen-positive adults, demonstrating the value of spatial analysis in post-MDA surveillance. Strategies to monitor cluster residents and high-risk groups are needed to reduce resurgence risk. Further research is required to quantify factors contributing to LF transmission at the last stages of elimination to ensure that programme achievements are sustained.

## Author Summary

Lymphatic filariasis (LF) is caused by infection with filarial worms that are transmitted by mosquito bites. Globally, 120 million people are affected, and 40 million are disfigured and disabled by complications such as severe swelling of the legs (elephantiasis). The Global Programme to Eliminate LF (GPELF) aims to interrupt disease transmission through mass drug administration (MDA), and to control illness and suffering in affected persons. In American Samoa, significant progress has been made towards LF elimination, and antigen prevalence has dropped from 16.5% in 1999 to <1% in 2011/2012 after seven rounds of MDA. Current challenges include identification of any residual hotspots of ongoing transmission, and effective strategies for early identification of any resurgence. Our study examined the prevalence and spatial distribution of LF antigens and antibodies in American Samoan adults to improve understanding of LF transmission in an area of low prevalence, develop tools and strategies to more accurately verify interruption of transmission, and provide evidence-based guidance for future elimination strategies in American Samoa.

## Introduction

Lymphatic filariasis (LF) is a neglected tropical disease of global importance, with an estimated 1.4 billion people in 73 countries at risk of infection. Over 120 million people worldwide are currently affected by lymphatic filariasis and 40 million are disfigured and disabled [1]. Infection is transmitted by mosquito vectors including *Anopheles*, *Aedes*, *Culex* and *Mansonia* species.

The Pacific Programme for Elimination of Lymphatic Filariasis (PacELF) was formed in 1999, and as part of the Global Programme to Eliminate LF (GPELF), aimed to eliminate the disease as a public health problem in 22 Pacific Island countries and territories (PICTs) by 2020 [2]. The Programme in the Pacific covers over 3000 islands and 8.6 million people, and consists of two strategies: firstly, to interrupt transmission through mass drug administration (MDA) using albendazole and diethycarbamazine (DEC) and secondly, to control morbidity and disability of affected persons [2]. Baseline surveys conducted in 1999 and 2000 determined that 11 PICTs were endemic for LF, five partially endemic, and six non-endemic [2]. Since then, variable progress has been made towards reducing prevalence and interrupting transmission on different islands [3], but significant success has been achieved in the Samoan Islands, particularly in American Samoa.

Before the 1960s, both Samoa (formerly called Western Samoa) and American Samoa had high prevalence (∼20%) of lymphatic filariasis [4], [5]. Multiple rounds of MDA in the 1960s had considerable impact and reduced the prevalence of microfilaraemia to less than 2%, but neither Samoa nor American Samoa managed to achieve sustained interruption of transmission at that time [6]–[9]. By 1999, antigen prevalence of 16.5% (N = 3018) was recorded in American Samoa and 4.5% (N = 7006) in Samoa. In American Samoa, after seven rounds of MDA from 2000–2006, antigen prevalence dropped to 2.3% (N = 1881) in 2007 in a community cluster survey that involved all age groups [10].

Current WHO guidelines [11] recommend that in areas where *W. bancrofti* is endemic and *Aedes* is the principal vector, the target threshold for post-MDA transmission assessment surveys (TAS) is <1% antigenaemia. Based on this target and sample sizes, critical cutoff values are calculated so that evaluation units have at least a 75% chance of passing if the true prevalence of antigenaemia is 0.5%, and no more than 5% of passing (incorrectly) if the true prevalence is ≥1%. For evaluation units where the number of antigen-positive individuals is below the critical cutoff value, no further MDA is recommended because of the low risk of continuing transmission. For areas where *Anopheles* or *Culex* is the principal vector, the target threshold is <2% antigenaemia.

American Samoa passed transmission assessment surveys (TAS) in 2011–12, designed to determine whether antigen prevalence using the ICT card test in 6 to 7 year old children was less than 1% [11]. The surveys found two ICT-positive children (N = 949, included 25 of 26 schools, critical cutoff of 6) on the main island of Tutuila and the adjacent island of Aunu'u, and 0% (N = 37, census at all schools) in the remote Manu'a islands [12]. In Samoa, TAS conducted in 2013 in three evaluation units (N = 3,585) found that while two units passed established targets, one unit (northwest Upolu) failed with 19 positives (N = 1,271, critical cutoff of 7) and further MDA was recommended [13].

The historical high risk of resurgence in the Samoan islands is likely to be related to a combination of factors, including poor MDA coverage and low compliance [14]; both day and night-biting mosquito vectors (including *Aedes polynesiensis* and *Aedes samoanus*) that are highly efficient at transmitting LF [15], [16]; and intense environmental drivers for transmission such as the tropical climate, high rainfall, abundance of suitable mosquito breeding sites, and outdoor lifestyle. Samoa and American Samoa were historically and ethnically one nation, divided into two separate political entities in 1899. There are continuing strong family, cultural, and economic links between the Samoan islands, with associated frequent high-volume travel (often for extended periods) and cross-migration. Reintroduction of parasites by infected travellers could therefore play an important role in potential resurgence [8], [17].

Previous studies in Samoa and Haiti found significant micro-spatial clustering of infection in areas of low prevalence, suggesting the potential for small residual foci of transmission at the neighbourhood scale even though overall average prevalence in an evaluation unit might be less than 1% [18], [19]. Such findings could potentially have significant implications for post-MDA surveillance strategies in certain epidemiological settings.

The WHO and GPELF have identified a number of knowledge gaps, key challenges and operational research priorities for LF elimination, including: i) the significance of residual microfilaraemia and antigenaemia in communities where the target threshold level has been achieved through MDA; ii) rapid identification of high-prevalence areas and development of strategies for dealing with them; and iii) development and standardization of cost-effective strategies for post-MDA surveillance [20]. While the initial phases of the programme focused mostly on developing guidelines, initiating and implementing country activities, and scaling up MDA rapidly to aim for full coverage, the latter phases require more attention towards ensuring a successful ending [21]. The sustained success of elimination programs depends on careful monitoring for potential resurgence post-MDA, particularly where there are residual foci with high prevalence, and where resurgence has historically proven to be a problem despite achieving low prevalence rates. The priorities in the endgame phase of LF elimination therefore include: i) intensive targeted studies of transmission thresholds, ii) new tools and strategies to accurately verify when transmission has been interrupted; and iii) effective post-intervention surveillance, monitoring, and evaluation to ensure timely detection of resurgence [21]. Additionally, as countries move closer to elimination, increasingly specific strategies and technical support are needed because of differences in local settings, needs, resources, constraints, and challenges [21]. Furthermore, as prevalence drops to very low levels after successful MDA, increasingly statistically robust surveillance strategies will be required to identify any ongoing transmission, particularly if confined to small geographic foci.

Currently, TAS typically include young children and do not provide any information on adults. Adults are the main reservoirs of any residual infections and are also susceptible to new infections, so surveillance of adults in the post-MDA phase of elimination programs could provide valuable information for identifying residual foci and detecting early resurgence. The current WHO guidelines encourage cost-efficient methods for post-MDA surveillance, such as the integration of LF surveillance activities with other population-based surveys as well as opportunistic screening of groups such as military recruits, hospital patients, and blood donors for microfilaraemia, antigenaemia, or antibodies [11]. Such activities will become increasingly important for GPELF as more countries reach elimination targets and move into the surveillance phase, but there is currently a paucity of evidence-based guidelines for conducting these activities or interpreting the findings.

We examined the seroprevalence of LF antigens and antibodies in American Samoan adults in 2010 to complement the results of TAS conducted in young children in 2011–2012, with the goal of providing a more complete picture of the status of LF in American Samoa after successful MDA. We used a serum bank and associated geo-referenced database to determine the seroprevalence of LF antigen and antibodies in adults, examine the spatial epidemiology of infection post-MDA, and identify any possible residual foci of infection and/or high-risk populations that might require targeted surveillance and monitoring. Our study aimed to address some of the knowledge gaps identified by WHO and GPELF by improving understanding of LF transmission in an area of low prevalence, explore the value of adult serological data for surveillance after successful MDA, develop new tools and strategies to more accurately verify interruption of transmission, and provide evidence-based guidance for future surveillance strategies in American Samoa.

## Methods

### Study location and setting

American Samoa consists of a group of remote islands in the South Pacific: the main island of Tutuila, the adjacent island of Aunu'u, and the remote Manu'a group of islands (Ta'u, Ofu, and Olosega). The census population in 2010 was approximately 56,000 [22], with over 90% residing on Tutuila, mostly in coastal villages. American Samoa has a tropical climate and is one of the wettest inhabited places in the world (average annual rainfall of over 3,000 mm), with rugged islands that include mountains, valleys, tropical rainforests, wetlands, fringing reefs, and lagoons.

*Wuchereria bancrofti* is the only filarial worm species found in American Samoa, and mosquito vectors include the highly efficient day-biting *Aedes polynesiensis* and night-biting *Aedes samoanus*.

### Serum bank and associated data

A serum bank was collected for a leptospirosis study in American Samoa in 2010 (four years after the last effective round of MDA for LF), and detailed description of the study design has been previously reported [23], [24]. Briefly, in Tutuila and Aunu'u, the study used a spatial sampling method that systematically selected households from a geo-referenced database of all houses on the islands. Sampling was designed to ensure maximum spatial dispersion over the study area to optimise geospatial analysis. In the very sparsely populated Manu'a Islands, the spatial sampling method was impractical, and non-random convenience sampling was used. The study included 807 adults (aged 18 to 87 years, 52.4% males) from 659 households in 55 villages on all five inhabited islands; 721 (89.3%) lived on the main island of Tutuila, and 555 (68.8%) had lived in American Samoa for their entire life. During the serum bank collection, the primary place of residence of each participant was geo-located using detailed village maps obtained from the American Samoa GIS User Group [25]. Questionnaires were used to obtain demographic data from participants, and were conducted by a team of interviewers who were fluent in both English and Samoan. The serum bank was highly representative of the adult population of American Samoa in both age and geographic distribution. Table 1 provides a summary of the demographics of the study population.

**Table 1:** Association between demographic variables and filarial antigen and antibodies.

	All participants		Og4C3>128				Og4C3>32				Wb123				Bm14			
	N	%	N	Prevalence	OR	*p*	N	Prevalence	OR	*p*	N	Prevalence	OR	*p*	N	Prevalence	OR	*p*
**Total samples**	807		805				805				806				806			
Total positive			6	0.7%			26	3.2%			65	8.1%			144	17.9%		
**Gender**																		
Females	380	47.1%	0	0.0%	-	-	8	2.1%	1		11	2.9%	1		41	10.8%	1	
Males	423	52.4%	6	1.4%	-	-	18	4.3%	2.1	0.1	54	12.8%	**4.90**	**0.00**	103	24.3%	**2.7**	**0.00**
**Age (years)**																		
<20	106	13.3%	0	0.0%	-	-	1	0.9%	1		3	2.8%	1		5	4.7%	1	
20–29	147	18.4%	2	1.4%	1	-	7	4.8%	5.3	0.12	12	8.2%	3.1	0.09	11	7.5%	1.6	0.38
30–39	142	17.8%	2	1.4%	1	0.98	7	4.9%	5.4	0.12	13	9.2%	3.5	0.06	31	21.8%	**5.6**	**0.00**
40–49	160	20.0%	1	0.6%	0.5	0.52	3	1.9%	2.0	0.55	16	10.0%	**3.8**	**0.04**	33	20.6%	**5.2**	**0.00**
50–59	119	14.9%	0	0.0%	-	-	5	4.2%	4.6	0.17	10	8.4%	3.1	0.09	30	25.2%	**6.8**	**0.00**
60–69	89	11.1%	1	1.1%	0.8	0.87	1	1.1%	1.2	0.90	8	9.0%	3.4	0.08	24	27.0%	**7.5**	**0.00**
>70	36	4.5%	0	0.0%	-	-	1	2.8%	3.0	0.44	3	8.3%	3.1	0.18	9	25.0%	**6.7**	**0.00**
**Years lived in AS**																		
Whole life	555	68.8%	4	0.7%			13	2.3%			39	7.0%			87	15.7%		
>10 years	718	89.0%	4	0.6%	1		19	2.6%	1		55	7.7%	1		129	18.0%	1	
5 to 10 years	54	6.7%	0	0.0%	-	-	2	3.7%	1.4	0.65	5	9.3%	1.2	0.67	6	11.1%	0.6	0.21
<5 years	28	3.5%	2	7.1%	**13.7**	**0.003**	4	14.3%	**6.2**	**0.002**	5	17.9%	2.6	0.06	8	28.6%	1.8	0.16
**Occupational groups**																		
Indoor	192	23.8%	0	0.0%	-		3	1.6%	1		9	4.7%	1		26	13.5%	1	
Outdoor	62	7.7%	0	0.0%	-		3	4.8%	3.2	0.16	6	9.7%	2.2	0.16	13	21.0%	1.7	0.17
Tuna cannery workers	73	9.0%	2	2.7%	3.3	0.17	4	5.5%	3.6	0.10	11	15.1%	**3.6**	**0.01**	16	21.9%	1.8	0.10
Others (mixed, unknown, unemployed)	480	59.5%	4	0.8%	1		16	3.3%	2.2	0.22	39	8.1%	1.8	0.12	89	18.5%	1.5	0.12
**Household income (USD)**																		
<$10,000	324	40.1%	3	0.9%	1		11	3.4%	1		30	9.3%	1		66	20.4%	1	
$10,000–$20,000	230	28.5%	3	1.3%	1.2	0.67	10	4.3%	1.3	0.55	20	8.7%	0.9	0.83	40	17.4%	0.83	0.39
$20,000–$30,000	61	7.6%	0	0.0%	-	-	0	0.0%	-	-	3	4.9%	0.5	0.28	12	19.7%	0.96	0.90
>$30,000	64	7.9%	0	0.0%	-	-	1	1.6%	0.5	0.45	2	3.1%	0.3	0.12	7	10.9%	0.48	0.08
Unknown	128	15.9%	0	0.0%	-	-	4	3.1%	0.9	0.89	10	7.8%	0.8	0.63	19	14.8%	0.68	0.18
					**Chi2/Fisher** *				**Chi2/Fisher** *				**Chi2/Fisher** *				**Chi2/Fisher** *	
**Island of residence**					*p*				*p*				*p*				*p*	
Tutuila	721	89.3%	6	0.7%	1.00		26	3.2%	0.10		57	7.1%	0.65		127	15.8%	0.62	
Other islands	86	10.7%	0	0.0%	1.00		0	0.0%	0.10		8	1.0%	0.65		17	2.1%	0.62	

Statistically significant results (*p*<0.05) highlighted in bold. OR = odds ratios on univariate logistic regression.

*Chi-squared or Fisher exact tests were used to compare differences between islands. Logistic regression was not performed because no antigen-positive cases were detected on the smaller islands.

### Ethics approval and informed consent

For the original leptospirosis study, approvals were obtained from the American Samoa Institutional Review Board (ASIRB), the Medical Research Ethics Committee of The University of Queensland (MREC-UQ), and the Queensland Health Forensic and Scientific Services Human Ethics Committee. The study was conducted in collaboration with the American Samoa Department of Health, and permissions for village visits were sought from the Department of Samoan Affairs and village chiefs and/or mayors. The study included only adult participants, all of whom provided written informed consent. For the current study, additional approvals to use the serum bank for lymphatic filariasis research were obtained from ASIRB and MREC-UQ.

### Serological analysis

All serological analyses were conducted at the WHO Collaborating Centre for Lymphatic Filariasis, Soil-transmitted Helminths, and other Neglected Tropical Diseases at James Cook University, Cairns, Australia. For all assays, sera were tested in duplicate. For any samples where the duplicates showed greater than 15% coefficient of variation in optical density (OD) reading, tests were repeated, as were those on plates with unsatisfactory standard curves or OD of less than 1.0 in the highest standard group. All plates were read using a VersaMax tunable ELISA reader (Molecular Devices) using Softmax Pro v5.3 software.

#### Og4C3 antigen ELISA test

This test detects circulating filarial antigen (CFA) in peripheral blood produced by adult filarial worms in the lympatics. ELISA kits from TropBio Pty Ltd, JCU, Townsville were used (now marketed by Cellabs Pty Ltd; www.cellabs.com.au) following manufacturer's instructions. Sera were diluted 1∶4 in sample diluent, and incubated overnight at 4 degrees Celsius. Known moderate positive (pool of ten samples from Papua New Guinea (PNG)) and negative sera (laboratory members who had never resided in LF-endemic areas) were included on each plate. A unit value for each sample based on a standard curve starting at an arbitrary 32,000 units with 4-fold dilutions. Two separate cutoff points were used: Og4C3 antigen >128 units (positive according to manufacturer's instructions), and >32 units (positive and equivocal). The mean value recorded for the negative sera over 20 plates was 4.9 units.

#### Wb123 antibody ELISA test

The test detects antibody to the Wb123 antigen identified from a library generated from L3 larval stages of *W.bancrofti* [26]. The assay was performed in ELISA format using plates pre-coated with 10 µg/mL Wb123 antigen. Sera at 1∶50 dilution in PBS/1%BSA/0.05% Tween 20 (PBS/T) were added at 50 µL per well, 50 µL of a known positive control were added at 1∶500 (high positive) and 1∶5000 (low positive) while negative control serum was added at 1∶50. Plates were incubated for 30 minutes at room temperature, washed 6 times in PBS/T and 50 µL per well of HRP conjugated mouse anti-human IgG4 (Invitrogen A10654) at 1∶5000 dilution was added for 45 minutes incubation. After 6 washes TMB substrate was added at 50 µL per well and the reaction was stopped with 1 M HCl after 5 minutes in the dark. Plates were read at 450 nm. Samples were classed as positive if their average optical density (OD) ratio was 9 or more times the negative control (based on the average ratio of of the low positive 1∶5000 serum control to blank). The mean OD ratio of the negative control to blank over 19 plates was 1.0.

#### Bm14 antibody ELISA test

The test detects antibody to an antigen identified from a cDNA library screened using sera from microfilaria positive people [27]. Sera were tested at 1∶50 dilution using the method described by [27] and [12]. A known positive control from PNG (S19) and negative serum from lab members were included on each plate. A 7-point standard curve in duplicate using a known high positive serum from PNG (S200) starting at 1∶200 dilution (1000 arbitrary units) and then 2–fold serial dilutions in PBS/T was included on each plate. A 4-parameter calibration curve was used to estimate the units of Bm14 antibody per sample. The cutoff for positivity was 125 units determined empirically as described by [27] using known positive and negative serum panels.

### Statistical analysis

Outcome measures used for statistical analyses were ELISA test results for each LF antigen and antibody. For Og4C3 antigen, statistical analyses were performed using two different cutoff points: >128 units (positive results) and >32 units (equivocal and positive results). Independent variables assessed included age, sex, years lived in American Samoa, occupation, household income, and island of residence. The number of years lived in American Samoa was categorized into <5 years (to reflect those who did not live in American Samoa during local MDA activities from 2000 to 2006), 5–10 years (those who lived in Am Sam during some of the local MDA activities), and >10 years (those who lived in Am Sam during all of the MDA activities). Occupation groups were categorized into those who worked i) predominantly indoors, ii) predominantly outdoors, iii) tuna cannery workers (the largest non-government employer in American Samoa; >90% of employees are migrant workers), and iv) others (including unemployed, unknown occupation, and those who have jobs that include both indoor and outdoor work). Data on household income was available in four categories. Island of residence was categorized into Tutuila and other islands.

The serum bank consisted of samples and data on 807 participants. There was sufficient serum in 805 samples to perform ELISA for Og4C3 antigen, and in 806 samples for Wb123 and Bm14 antibodies. Data on gender were available for 803 participants, on age for 798, on years lived in American Samoa for 800, and on household income for 679. Island of residence and geo-locations of households were available for all participants.

Chi-squared or Fisher exact tests were used to compare outcomes for categorical independent variables. Variables with *p*<0.1 were selected for further analyses using univariate logistic regression, and odds ratios (OR) were calculated. STATA v11.1 software (StataCorp, College Station, Texas) was used for all analyses, and *p* values of <0.05 were considered to indicate statistical significance.

### Analysis of geographical clustering of serological indicators

During the serum bank collection, the primary place of residence of each participant was geo-located using village maps produced using geo-referenced data from the American Samoa GIS User Group [25]. Data available included island/village boundaries, and the location of houses, schools, churches and major infrastructure. For spatial analyses, only data from the main island of Tutuila were included. Populations and inhabited areas on Aunu'u and the Manu'a islands were too small for geospatial analysis to be meaningful. Maps were produced to show the distribution of participants' households, and locations of participants with positive ELISA for each antigen and antibody. The spatial distribution of participants based on years lived in American Samoa was also examined to determine if migrants were concentrated in any villages. All geo-spatial data were collated, stored, linked and mapped using ArcMap v10.0 (Environmental Systems Research Institute, Redlands, CA).

Spatial dependence in the positive serological results for each antigen and antibody was investigated using a semivariogram in the statistical software R, using the geoR package version 2·14·1 (The R foundation for statistical computing). A semivariogram is a graphical representation of the spatial variation which allows for the quantification of spatial cluster size and the tendency for geographical clustering within a region. The semivariogram is characterized by three parameters: the sill, which is the spatially structured component of the semivariance (indicative of the tendency for geographical clustering); the nugget, which is the spatially unstructured component of the semivariance (representing random variation, very small-scale spatial variability or measurement error); and the range, which is the distance at which locations can be considered independent (indicative of the size of geographical clusters). To estimate the proportion of the variation that was spatially structured we divided the partial sill by the sum of the partial sill and nugget.

## Results

### Seroprevalence

Og4C3 antigen levels of >128 units (positive result) were found in 0.75% (6 persons, 95% CI 0.3–1.6%) of participants, and levels of >32 units (equivocal plus positive results) in 3.2% (26 persons, 95% CI 0.6–4.7%). The seroprevalence of Wb123 and Bm14 antibodies were 8.1% (65 positives, 95% CI6.3–10.2%) and 17.9% (144 positives, 95% CI 15.3–20.7%) respectively.

### Factors associated with positive LF antigen and antibodies

**Figure 1:**
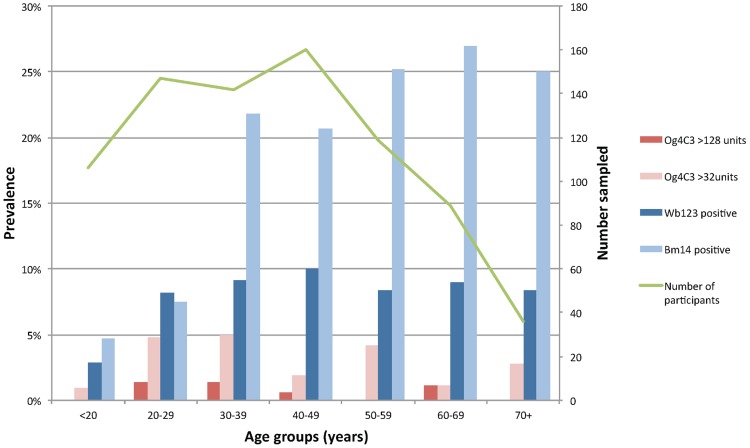
Prevalence of filarial antigen and antibodies by age groups, American Samoa 2010.

Table 1 provides a summary of the associations between demographic variables and the presence of LF antigen and antibodies. Our results show that both antigen and antibody prevalence were higher in males compared to females (Table 1). Figure 1 shows the age distribution of participants, and the prevalence of antigen (Og4C3>128 and Og4C3>32) and antibodies (Wb123 and Bm14) in each age group. Antigen-positive individuals were identified in all age groups, with no significant difference between ages. Prevalence of both Wb123 and Bm14 antibodies were higher in the older age groups. In participants aged 30 years and older, Bm14 prevalence was two to three times higher than Wb123 prevalence in all age groups.

**Figure 2:**
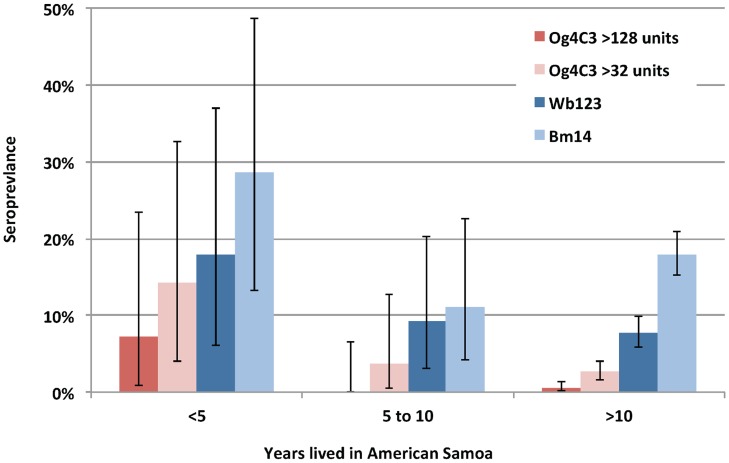
Prevalence of filarial antigen and antibodies by years lived in American Samoa.

Antibody and antigen prevalence were inversely associated with the number of years lived in American Samoa (Figure 2 and Table 1). Of all study participants, 68.8% (n = 555) had lived in American Samoa for all of their lives. Compared to individuals who had lived in American Samoa for over 10 years, new migrants who had lived there for <5 years had odds ratios of 13.7 (95% CI: 2.4–78.4) of having Og4C3 antigen of >128 units, and odds ratio of 6.1 (95% CI: 1.9–19.4) of having Og4C3 antigen of >32 units (Table 1). New migrants also had higher prevalence of Wb123 and Bm14 antibodies compared to those who had lived in American Samoa for >10 years, but differences were not statistically significant. The prevalence of antibodies and antigen were higher in residents on the main island of Tutuila compared to those who lived in smaller islands, but differences were not statistically significant. Tuna cannery workers had significantly higher prevalence of Wb123 antibodies, but there were no other associations between occupational groups and seroprevalence. Our study did not find any association between income and seroprevalence.

### Geographical clustering of serological indicators

**Figure 3:**
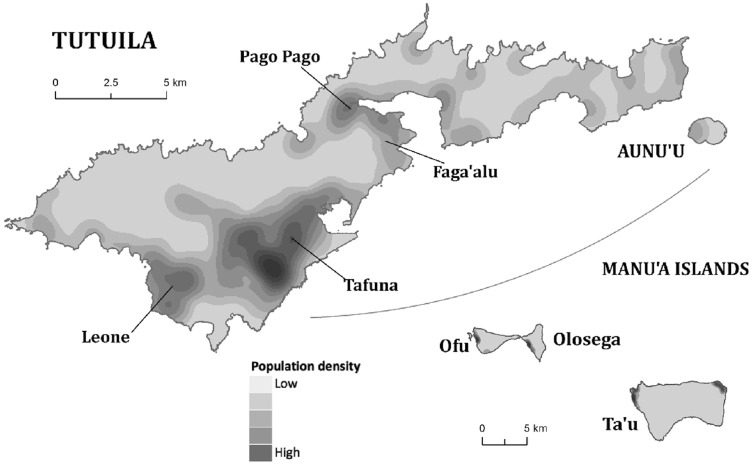
Population distribution on the islands of American Samoa 2010 (Reproduced from Lau et al. (23).

**Figure 4:**
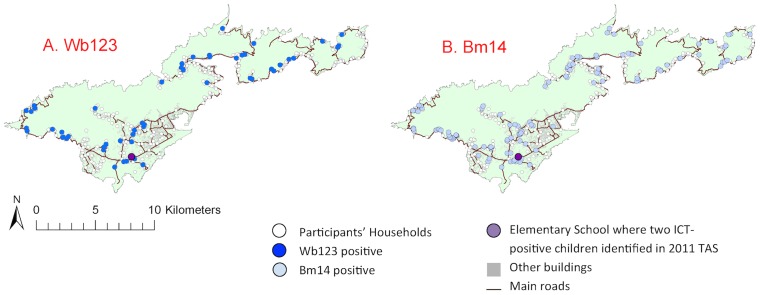
Household locations of individuals with positive and negative antibodies on Tutuila. A. Wb123, B. Bm14.

**Figure 5:**
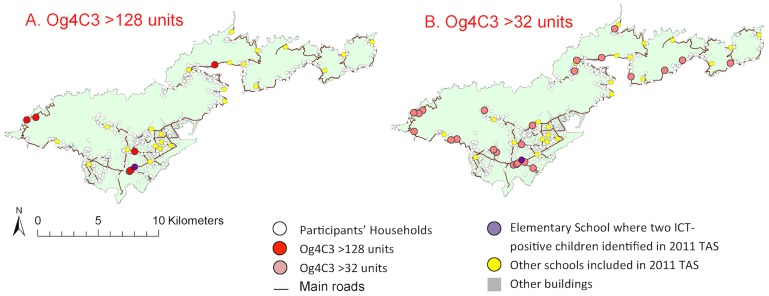
Household locations of individuals with positive and negative antigen on Tutuila. A. Og4C3>128 units, B. Og4C3>32 units.

**Figure 6:**
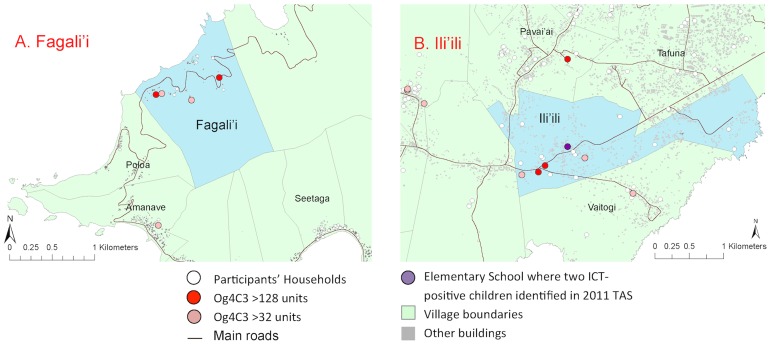
High resolution village maps of A. Fagali'I and B. Ili'ili, showing household locations of individuals with Og4C3 antigen of >128 units and >32 units, and school where two ICT-positive children identified in 2011 TAS.

For reference, a kernel density map of population distribution in American Samoa is shown in Figure 3 (reproduced from [23]). The household locations of individuals with positive and negative Bm14 and Wb123 antibodies are shown in Figure 4a and 4b, and positive/equivocal Og4C3 levels shown in Figures 5a and 5b. High resolution maps of the villages of Fagalii (Figure 6a) and Ili'ili (Figure 6b) show the locations of participants' households, those with positive/equivocal results for Og4C3, and the location of the elementary school where two ICT-positive children were identified during the 2011 Transmission Assessment Survey.

**Figure 7:**
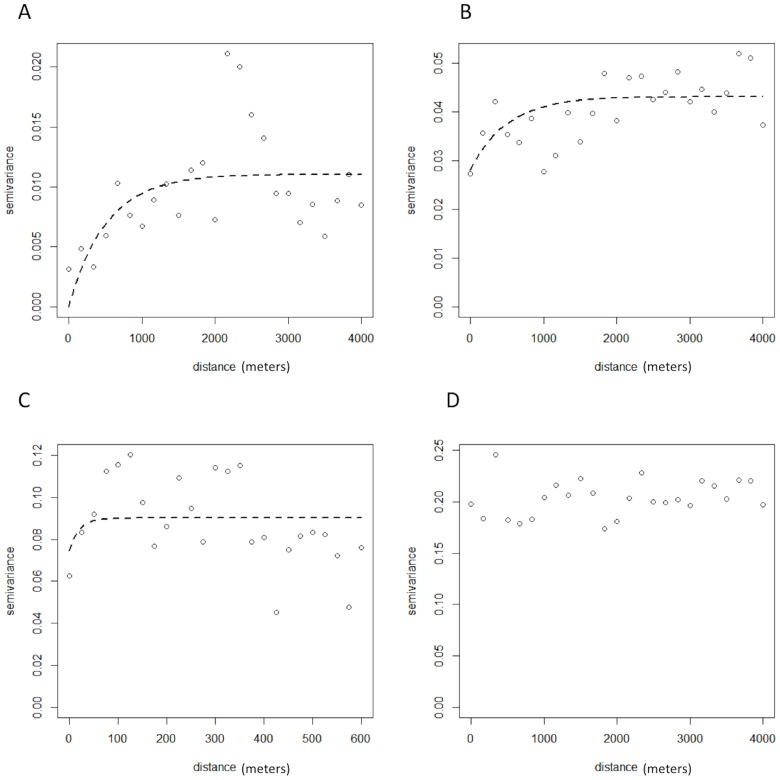
Semivariograms of spatial dependence of antigen and antibodies: A. Og4C3>128 units, B. Og4C3>32 units, C. Wb123 positive, D. Bm14 positive.

While the semivariograms for Wb123 and Bm14 antibodies did not reveal any significant small-scale spatial variation, the semivariograms for antigen (both Og4C3>128 units and Og4C3>32 units) showed considerable residual spatial variation (Figure 7 and Table 2). Our results indicate that the average size of a cluster for Og4C3>128 units was 1,242 metres and the proportion of the variation in Og4C3>128 units explained by geographical proximity was 85%. The average size of a cluster for Og4C3>32 units was 1,498 meters and the proportion of the variation in Og4C3>32 units explained by geographical proximity was 62%. Migrants who had lived in American Samoa for <5 years and 5–10 years were dispersed throughout the territory, and no significant clustering was found.

**Table 2:** Spatial parameters of geographical clustering of Og4C3 antigen, and Wb123 and Bm14 antibodies.

Spatial parameters	Og4C3>128	Og4C3>32	Wb123	Bm14
Range (meters)	1,242	1,498	60	NA
Partial sill	0.00965	0.0451	0.015	NA
Nugget	0.00173	0.0281	0.075	NA
Proportion of variance due to spatial dependence (%)	85	62	17	NA

## Discussion

Our study demonstrates that high-risk populations for LF in American Samoa include adult males and recent migrants. The results also suggest the possible existence of residual foci of antigen-positive individuals in American Samoa. Although our findings do not provide conclusive evidence of recent transmission, further investigation is recommended to confirm (or otherwise) the possible high-risk populations and locations, and determine whether ongoing targeted surveillance of these groups is warranted, particularly in the Samoan Islands where there is a history of resurgence despite achieving very low prevalence [4].

The prevalence of Wb123 and Bm14 antibodies differed significantly, and further research is required to understand the role of each laboratory test in post-MDA surveillance. There was a sharp rise in Bm14 antibody prevalence from age 30–39 years, which was also observed by Mladonicky et al in 2006 in three villages of American Samoa [9]. We found that Wb123 antibody prevalence peaked in participants aged between 30 and 40 years, but at much lower prevalence than Bm14 antibody. Wb123 antibody is a relatively new assay, and the indicative cutoff point used in this study could have contributed to the differences between the prevalence of Wb123 and Bm14 antibodies. Neither Wb123 nor Bm14 antibody prevalence declined with age, but at present we cannot distinguish long-term persistence of antibodies from ongoing transmission. Other studies have noted persistence of Wb123 antibodies in adults for many years after MDA, although significant decline was observed in those who were antigen-negative [28].

Positive Og4C3 antigen was found in all age groups and did not show any age-specific patterns. The presence of Og4C3 is not necessarily associated with microfilaraemia and does not provide evidence of ongoing transmission. Antigen prevalence drops dramatically after MDA, but it is not possible to unequivocally distinguish between recent or past infection based on Og4C3 alone. However, for *W. bancrofti* areas, the WHO currently supports the use of circulating filarial antigen prevalence (measured by ICT card test) as an indicator of LF infection, and it is one of the options of diagnostic tests used for measuring the prevalence of infection at each stage of the elimination process (pre-MDA mapping, sentinel and spot check sites, and TAS). The Og4C3 antigen has also been used in a similar study in Haiti that investigated clustering of residual antigen-positive persons in low endemic areas [19].

In our study, three aspects of the Og4C3-positive individuals raised suspicion about the possibility of recent transmission. Firstly, one cluster of Og4C3-positive adults was located in very close proximity to the two ICT-positive children found during the 2011 TAS. Secondly, Og4C3 prevalence in our study was higher in migrants (mostly from Samoa) even though baseline antigen prevalence in 1999 was much lower in Samoa (4.5%) than American Samoa (16.5%). If positive Og4C3 in our sample predominantly reflected infections in the remote past, prevalence would be expected to be lower in the migrants. Thirdly, we found significant spatial clustering of Og4C3 antigen, but not of Wb123 or Bm14 antibodies. If the Og4C3-positive adults in our study were predominantly infected in the remote past, clustering would have been much less likely, as demonstrated by the absence of clustering of antibody-positive adults.

Data on microfilaraemia would have helped determine the presence of ongoing transmission, but this was not possible with a serum bank. Despite this limitation, we believe that our seroprevalence study of adults provided valuable information about potential residual infections in American Samoa. Similar studies should be considered elsewhere for post-MDA surveillance and for identifying high-risk populations and/or locations that might warrant more intense targeted surveillance.

Higher LF seroprevalence in males corroborates findings from some of the previous studies in Samoa [7], [18] and American Samoa [29], and could be explained by more time spent outdoors for work and recreation compared to females. Interestingly, LF prevalence was found to be equivalent in males and females in 1999 prior to MDA in American Samoa, but Liang et al. reported a shift toward higher prevalence in males in sentinel site surveys conducted during and after MDA [29]. Our study (using a much larger and more representative sample of the adult population) confirms the higher prevalence among males post-MDA in American Samoa.

Our results also indicate higher antigen prevalence in new migrants, who were mostly from a neighbouring LF-endemic country where transmission is still occurring in some areas. This suggests that human movement could be an important pathway for parasite reintroduction and subsequent resurgence of LF in American Samoa. Visitors and migrants travel for family, work, and economic reasons and usually live and work in close proximity to local American Samoans. Prolonged visits and cross migration are also common, and further increase the chances of parasite reintroduction. In addition, American Samoans also travel frequently to Samoa and other neighbouring Pacific Islands, and could be at significant risk of infection if staying for extended periods in areas of high prevalence. In 2012, there were a total of 67,979 international arrivals to American Samoa (with a local population of ∼56,000). Of these 44,830 were citizens of other Pacific Islands, including 22,600 arrivals of returning citizens of American Samoa. A total of 20,082 arrivals were Samoan citizens, with 158 travelling for business, 4,158 for employment, 7,123 returning residents, and 8,757 visiting relatives [22]. Further research is required to improve understanding of the role of human movement in parasite reintroduction into American Samoa, and the consequent risk of resurgence based on travel patterns between Samoa and American Samoa, and LF prevalence at places of origin of visitors and new migrants. Cross-border strategies to coordinate efforts between Samoa and American Samoa for LF elimination and surveillance should also be considered.

American Samoa's population mostly live on ancestral land, and most of the study participants had lived in the same village for most or all of their lives, thus providing an excellent opportunity to examine disease transmission patterns. Our results indicate that most of the spatial distribution of antigenaemia could be accounted for by geographical proximity of place of residence. Geo-spatial analysis provided some evidence of possible micro-spatial clustering of antigen-positive adults at the neighborhood level at two villages. Clustering at the household level suggests that the home environment is important in transmission even though one of the major vectors is day-biting. The close proximity between the elementary school attended by the two ICT-positive young children identified during the 2011 TAS and one of the possible village clusters suggests possible ongoing transmission. Our results indicate an average cluster size of 1,200 meters to 1,500 meters for antigenaemia, and the estimate of cluster size provides important information for the design of further studies to identify local transmission foci.

Our study demonstrates the potential value of geospatial databases in post-intervention surveillance, monitoring, and evaluation for identifying possible micro-spatial clusters that might not be captured by routine TAS alone. Early detection of such clusters could be essential for timely intervention to reduce the risk of resurgence. Geospatial analysis could therefore potentially be used as an additional tool for verifying elimination status and for confirming that transmission has been interrupted. Changes in the spatial distribution of serological markers over time would also potentially be useful for identifying focal transmission, but unfortunately results of previous surveys in American Samoa were only located to the village rather than household level, and not of sufficiently high spatial resolution for the types of analyses conducted in this study or for comparing changes over time.

Further operational research could also explore the use of geospatial data for informing programme delivery (e.g. by identifying the size of clusters and delineating areas that might warrant targeted surveillance and monitoring); calculating the distance of influence on infection risk that antigen-positive persons have on their near neighbours; and determining transmission threshold targets that include a spatial component rather than just a simple average prevalence for an entire evaluation unit. The accuracy of prevalence estimations in evaluation units will also depend on spatial heterogeneity within the boundaries of the unit. Risk of LF and drivers of transmission are unlikely to be entirely uniform within any evaluation units, and be determined by many factors such as climatic conditions, population density, urban versus rural areas, MDA coverage, and vector species and density. The average prevalence in an evaluation unit could therefore mask focal areas of high prevalence (hotspots) if they are surrounded by large areas of low prevalence. Consequently, estimations of average prevalence in an area could vary greatly depending on how evaluation units were determined. Hotspots are more likely to be missed if they are small, in evaluation areas with greater spatial heterogeneity in risks and drivers, and when prevalence is very low such as in the post-MDA surveillance phase. Careful definition of evaluation units will therefore be crucial for optimising the probability of identifying any residual hotspots of transmission or early resurgence.

One of the challenges pertaining to geospatial methods of cluster detection when utilising point location data is that such data are prone to random error and random variation in the presence of rare disease events and/or inadequate representation of the population at risk. We therefore used a robust geostatistical method to identify the presence of geographical clustering in our point location data by partitioning the variation in data that was due to random error and the variation that is due to spatial clustering. Semivariography (as utilized in this study) demonstrated that spatial clustering was present in the study area (Tutuila) but does not identify the location of clusters. The location of clusters could be further investigated by using model-based geostatistics that account for diagnostic uncertainty and variation in factors such as climate, population, and entomological parameters to produce predictive risk maps of LF.

Spatial decision support systems are being used for malaria elimination programs, and similar tools could also be useful for LF [30]. A geospatial platform could also be used to integrate environmental and entomological data with human surveillance data, and used to explore possible environmental drivers of disease transmission, the impact of vector control on elimination programs, and the potential for using xenomonitoring to enhance post-MDA surveillance.

This study also demonstrates the usefulness of high-quality serum banks for investigating multiple diseases (a dengue seroprevalence study was also conducted using the same serum bank [31]), and provides an example of successful collaboration between researchers of different diseases to improve the cost-effectiveness of field epidemiology investigations, which are often expensive and logistically challenging. We believe that the WHO's recommendations of integrating of LF surveillance activities with other population-based surveys are logistically feasible and practical.

Our findings should be interpreted in light of potential limitations. First, the serum bank used for the study was collected for a leptospirosis study, and we could not ascertain whether participants had previously been diagnosed with or treated for LF, or participated in MDA in American Samoa or elsewhere. Only 28 participants (3.9%) were recent migrants (lived <5 years in American Samoa), and although they would not have been living in American Samoa during MDA activities, some might have received MDA in Samoa or other home countries. However, we have no reason to believe that our participants were biased with respect to MDA compliance locally or elsewhere. Second, there were only six participants with Og4C3 of >128 units, and 26 participants with Og4C3 of >32 units, and small numbers could have affected the accuracy of spatial analyses. Small numbers generally reduce the likelihood of identifying statistically significant associations, but despite this, we found significant results using robust tests and geospatial analyses. Third, participants in the serum bank included adults of all ages, but did not include children or adolescents. Results of TAS conducted at about the same time provided antigen prevalence data in 6–7 year old children, but data on antigen and antibody levels in children of all ages would help improve understanding of the application of diagnostic tests for post-MDA surveillance. Finally, participants were geo-located to place of residence, but LF infection could occur elsewhere, particularly in the presence of efficient day-biting vectors. If vectors were predominantly night-biting, clustering of infections could potentially be even more readily defined around household locations.

This study provides preliminary results to support the importance of further research designed to specifically focus on improving understanding of disease transmission at the last stages of elimination when prevalence is very low; answering operational questions in LF elimination programs, especially the role of migration; developing tools to enhance the effectiveness of post-MDA surveillance and monitoring; and providing an evidence base for elimination strategies and targets. Follow up studies are being conducted in American Samoa to determine whether hotspots truly exist, develop models to quantify the significance of migrants in LF elimination, and explore the use of molecular xenomonitoring in the Pacific Island setting. The study also highlights the importance of assessing locally relevant risks for infection, which could vary significantly between places depending on cultural, societal, and environmental factors, as well as filarial species and mosquito vectors. The approach and results of this study are specifically relevant for the Samoan islands, but could also provide insight into LF transmission in other LF-endemic areas, and be pertinent to other Pacific Islands with similar vectors, lifestyle, culture, climate, environmental conditions, and migration patterns.

## Supporting Information

Checklist S1 STROBE Checklist for observational studies.
(DOC)
